# Xanthogranulomatöse Adrenalitis: Seltene und schwierige Differentialdiagnose von Nebennierentumoren

**DOI:** 10.1007/s00292-024-01312-x

**Published:** 2024-03-12

**Authors:** Wolfgang Saeger, Andreas M. Luebke, S. T. Mekoula, Jörg-Michael Pahnke

**Affiliations:** 1 Institut für Pathologie des Universitätsklinikums Hamburg-Eppendorf, Universität Hamburg, UKE, Martinistr. 52, 20246 Hamburg, Deutschland; 2 Klinik für Urologie und Kinderurologie des St. Barbara-Hospital, Gladbeck, Deutschland; 3 Pathologisches Institut, Recklinghausen, Deutschland

**Keywords:** Histiozytom, Erdheim-Chester-Krankheit, Immunhistologie, Malakoplakie, Langerhans-Zellhistiozytose, Histiocytoma, Erdheim–Chester disease, Immune histology, Malakoplakia, Langerhans cell histiocytosis

## Abstract

Ein radiologisch festgestellter Tumor einer 29-jährigen Frau mit Fieber um 39 °C wurde unter dem Verdacht einer Cholezystitis oder eines Leberabszesses operiert, dabei ein solider Tumor in der Nebennierenloge gefunden und reseziert. Die Schnellschnittbefundung ergab keine klare Diagnose bezüglich der Dignität und der Zuordnung. Histologisch zeigte sich, dass der Tumor aus dicht gelagerten großen histiozytenartigen Zellen mit Expression von Vimentin, CD68 und CD163 sowie Negativität für Keratin, Langerin und SMA aufgebaut ist. Wir diagnostizierten eine xanthogranulomatöse Adrenalitis und diskutierten die Differentialdiagnosen (Langerhans-Zellhistiozytose, Rosai-Dorfman-Krankheit, Malakoplakie, Erdheim-Chester-Krankheit).

## Abstract (en)

A radiologically diagnosed tumor in a 29-year-old woman with a fever of around 39 °C was operated on under the suspicion of cholecystitis or a liver abscess. A solid tumor was found in the adrenal gland and resected. The frozen section findings did not reveal a clear diagnosis of entity and assignment. Histologically, the tumor was found to consist of densely clustered large histiocyte-like cells with expression of vimentin, CD68, and CD163 as well as negativity for keratin, langerin, and SMA. We diagnosed xanthogranulomatous adrenalitis and discussed the differential diagnoses (Langerhans cell histiocytosis, Rosai–Dorfman disease, malakoplakia, Erdheim–Chester disease).

Radiologisch wurde ein 15 cm durchmessender Tumor in der rechten Nebennierenloge festgestellt. Die Tumorresektion entfernte einen histiozytären, nicht neoplastischen Tumor, der pathohistologisch und trotz umfangreicher Immunhistologie große differenzialdiagnostische Schwierigkeiten bereitete und letztendlich als xanthogranulomatöse Adrenalitis klassifiziert wurde. Die verschiedenen differentialdiagnostisch in Frage kommenden Entitäten werden diskutiert.

## Fallbericht

Eine 29 Jahre alte, zuvor gesunde Frau wurde wegen einer seit 10 Tagen bestehenden Obstipation mit Appetitlosigkeit, rechtsseitigen epigastrischen Schmerzen und Fieber bis 39 ^°^C sowie einer hausärztlich sonographisch nachgewiesener Raumforderung im Morrison-Pouch stationär aufgenommen. Im CT ergab sich eine inhomogene solide, malignomverdächtige Raumforderung am Oberpol der rechten Niere von 11,7 × 9,1 × 10,5 cm mit nicht sicherer Abgrenzung zur rechten Niere und Leber sowie Kontakt zur V. cava inferior. Auffällige Laborwerte waren eine deutliche Vermehrung der Thrombozyten, eine Erhöhung des C‑reaktiven Proteins (143 mg/l), der alkalischen Phosphatase (203 U/l) und der Gamma-GT (98 U/l). Eine Hormondiagnostik erfolgte nicht. Als klinische Diagnose wurde der Verdacht auf eine komplizierte Nierenzyste, differenzialdiagnostisch eine akute Cholezystitis oder ein Leberabszess gestellt.

Die Operation erfolgte zur Diagnostik der unklaren tumorösen Läsion mit parakolischer Inzision des parietalen Peritoneums und Mobilisation des Colon ascendens nach medial mit Darstellung der Niere. Der Tumor erschien schlecht abgrenzbar, verwachsen und nicht zystisch sondern solid. Die Punktion ergab keine Flüssigkeit. Die Niere wurde freigelegt und der Tumor vom Leberunterrand, der V. cava und von der Niere abpräpariert und geborgen. Die Schnellschnittuntersuchung ergab einen Tumor unklarer Dignität und unsicherer Herkunft. Der postoperative Verlauf war komplikationslos.

## Material und Methode

Das Operationspräparat wurde in üblicher Weise gemessen, gewogen, zerlegt und in 10 % gepuffertem Formalin fixiert. Die Paraffinschnitte wurden mit HE, PAS und EvG gefärbt. Die eingesetzten immunhistologischen Marker verzeichnet die Tab. 1.

**Table 1:** 

Marker	Ergebnis XA	Ergebnis NNR
Keratin AE1/AE3	Negativ	1+
Vimentin	3+	Negativ
S100 Protein	(+)	Negativ
CD 10	(+)	Negativ
EMA	1+	Negativ
SF‑1	Negativ	3+
CK 18	(+)	Negativ
CD 163	3+	Negativ
CD 68	3+	Negativ
CD 1a	Negativ	Negativ
Langerin (CD 207)	Negativ	Negativ
Atypische Lymphozyten Kinase (ALK)	(+)	Negativ
Glattmuskuläres Aktin	Negativ	Negativ
Desmin	Negativ	Negativ
CD 34	Negativ	Negativ
Stat6	Negativ	Negativ
Inhibin	Negativ	1+
Melan A	Negativ	3+
IgG 4	Negativ	Negativ
IgG	Negativ	Negativ
Ki-67	5–20 %	Negativ

*XA* xanthogranulomatöse Adrenalitis, *NNR* Nebennierenrinde

## Makroskopische Befunde

Das Operationspräparat wog 636 g und maß 145 × 115 × 90 mm und schloss die rechte Nebenniere ein. Die Oberfläche erschien kapselartig mit Verwachsungen. Die Schnittflächen zeigten in der Peripherie einen schmalen Saum gelblichen Nebennierenrindenrestparenchyms. Der darunter liegende Tumor erschien mäßig fest, solide, bräunlich, leicht glasig und ohne Nekrosen oder Zysten.

Durch den ihn umgebenden Nebennierensaum konnte er eindeutig der Nebenniere zugeordnet werden.

## Mikroskopische Befunde

**Figure 1:**
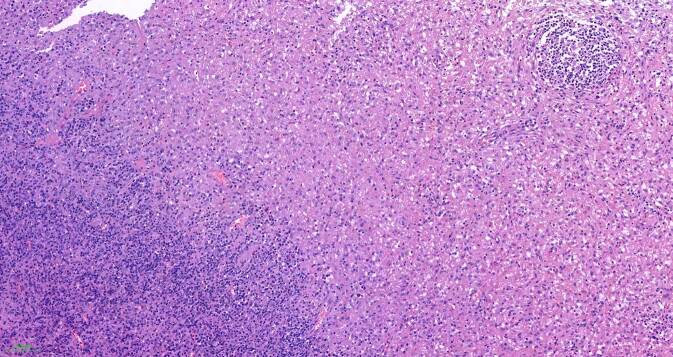

**Figure 2:**
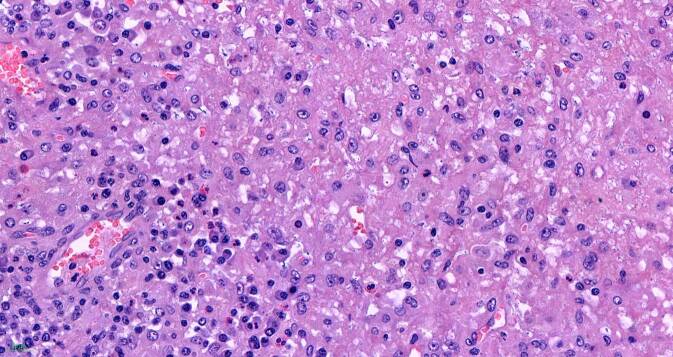

**Figure 3:**
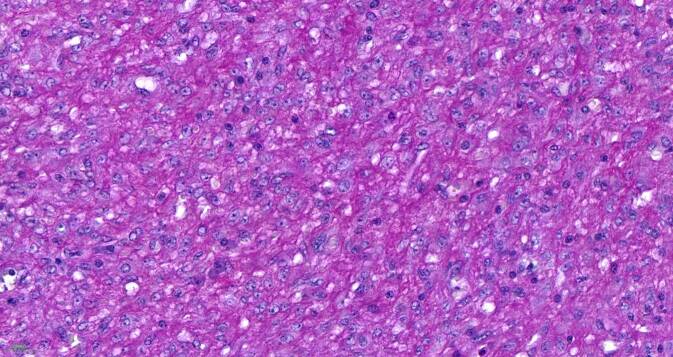

**Figure 4:**
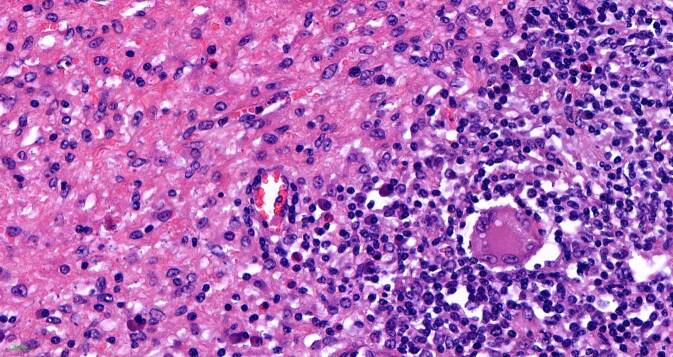

**Figure 5:**
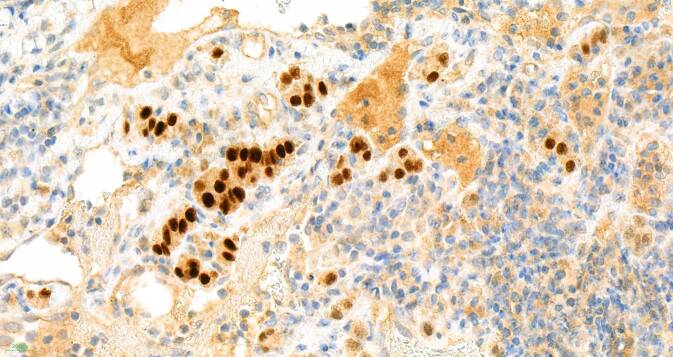

Die tumorartige Läsion zeigt eine ausgedehnte diffuse Proliferation aus relativ großen, monomorphen histiozytären Zellen (Abb. 1). Ihre Kerne sind gleichförmig rund bis rundoval und Chromatin-arm. Die Nukleolen sind klein (Abb. 2). Das recht breite Zytoplasma ist schwach eosinophil und PAS-positiv (Abb. 3). Die Zellmembranen sind undeutlich. Zwischen den Zellen ist etwas Fasergewebe septenartig oder feinnetzig entwickelt. Fokal kommen mehrkernige histiozytäre Riesenzellen zur Darstellung (Abb. 4). Begleitend zeigt sich ein gemischtes Entzündungsinfiltrat aus Lymphozyten und Plasmazellen sowie eingestreuten Mastzellen, neutrophilen und eosinophilen Granulozyten. Pilze sind in der PAS-Färbung nicht identifizierbar. Stellenweise unscharf begrenzt sieht man Nebennierenrindenrestgewebe mit mehr spongiozytären als kompakten Zellen mit immunhistochemisch nukleärer Positivität für SF1 (Abb. 5). Nebennierenmarkgewebe war nicht nachweisbar.

Die immunhistologischen Befunde verzeichnet die Tab. 1. Sie führten zum Ausschluss eines primär adrenokortikalen (SF-1- und Melan-A-negativ) oder adrenomedullären (Synaptophysin- und Chromogranin-negativ) Tumors und einer Karzinommetastase (Keratin-negativ).

Die Struktur erschien gut vereinbar mit einer histiozytenreichen granulomatösen Läsion, gut passend zu einer xanthogranulomatösen Adrenalitis (XA).

## Diskussion

Tumorartige histiozytäre Prozesse der Nebenniere sind sehr selten. Da die Langerhans-Zellhistiozytose in der Nebenniere sehr wahrscheinlich nicht vorkommt [1] und die läsionalen Zellen negative Färbereaktionen gegenüber S100, CD1a und Langerin zeigten (Tab. 1), kann die Differenzialdiagnose auf Non-Langerhans-Zellhistiozytosen beschränkt werden, die in der Literatur beschrieben sind (Tab. 2).

**Table 2:** 

Marker	Erdheim-Chester-Erkrankung [4, 7]	Rosai-Dorman-Erkrankung [3]	Xanthomatöse Adrenalitis [2, 5, 6]	Malakoplakie [8]
Altersgipfel	40–60 Jahre	Kinder und junge Erwachsene	50–60 Jahre	Median 50 Jahre
Geschlecht/ Risikopatienten	Leichtes Überwiegen der Männer	Assoziiert oft mit viralen oder tumorösen Erkrankungen	Männer	Häufiger Frauen
Lokalisationen	Lange Röhrenknochen, ZNS, Skelett, Lunge, Herz, Lymphknoten, Retroperitoneum, Nebenniere, perirenales Weichgewebe	Lymphknoten, Niere, Haut, Nebenniere	Nebenniere, evtl. zusätzlich paraadrenal	Häufig im Harntrakt: Niere, Harnleiter, Harnblase, seltener Gastrointestinaltrakt, genital, pulmonal, dermal und adrenal
Histologie	Xanthomatöse Infiltration	Große Histiozyten mit eosinophilem granuliertem Zytoplasma, Lymphozyten, Plasmazellen	PAS-positive lipidreiche Makrophagen, Plasmazellen, Lymphozyten, Granulozyten, Fremdkörperriesenzellen	Michaelis-Gutmannn-Körper mit Positivität in PAS, von Kossa- und Fe-Färbung
Immunhistologie	Positiv: CD68, CD163, CD14, Faktor XIIIa negativ: S100-Protein, CD1a, Langerin	S100 positive Histiozyten	Positiv für CD68 und Vimentin, negativ für epitheliale Marker	Positiv für CD68

Der fehlende Nachweis charakteristischer schaumzelliger Makrophagen, das junge Patientenalter sowie der fehlende Hinweis auf eine knöcherne Beteiligung machen eine Erdheim-Chester-Erkrankung weitestgehend unwahrscheinlich.

Hinweise auf eine extranodale Rosai-Dorfman-Erkrankung ergeben sich ohne konventionell histologisch sichtbare Emperipolese und fehlener immunhistochmischer Positivität der Histiozyten für S100 nicht.

Die für eine Malakoplakie charakteristischen Michaelis-Gutmann-Körper sind nicht nachweisbar.

Ein adrenales Neoplasma ist sowohl konventionell histologisch und bei Negativität aller immunhistochemischen Nebennierenmarker ausgeschlossen, ebenso wie die Metastase eines epithelialen Tumors.

In der Literatur finden sich weitere Einzelkasuistiken zur xanthomatösen Adrenalitis. In dem Fall von Atiemo et al. [2] wird eine sehr schaumzellreiche Adrenalitis beschrieben, die 15 Monate nach Entfernung eines Renalzellkarzinoms auf der Gegenseite sich entwickelt hatte und die als Folge des zurückliegenden Eingriffs angesehen wurde. Bei einem weiteren Fall [6] wurden bei einem Diabetiker ebenfalls reichlich Schaumzellen, aber auch erhebliche akute wie chronische entzündliche Infiltrate sowie Methicillin-resistente Bakterien vom Streptococcus aureus nachgewiesen. Beide Fälle unterscheiden sich somit deutlich von unserem Fall, bei dem keine Ursache gefunden wurde und die Struktur der Histiozyten nur stellenweise angedeutet schaumzellig erschien, aber eine Fiebersymptomatik auf ein entzündliches, wenn auch nicht sicher bakterielles Geschehen hinwies.

## Fazit für die Praxis

- Zusammenfassend konnten wir zeigen, dass die xanthogranulomatöse Adrenalitis eine seltene, aber wichtige Differentialdiagnose adrenaler Tumoren ist, welche sich jedoch erst am Operationspräparat offenbarte.
- Ferner kann die Abgrenzung gegenüber Histiozytosen erhebliche Schwierigkeiten bereiten, welche eine sorgfältige Differenzierung des entzündlichen Zellinfiltrates erfordert.
- Zusätzlich sichert die abschließende Korrelation mit Klinik und Bildgebung die diagnostische Einordnung.
- Im vorliegenden Fall ist die klinische Symptomatik mit Fieber und Verdacht auf Cholezystitis/Leberabszess im Einklang mit der Diagnose einer xanthogranulomatösen Adrenalitis.
